# Assessing Practice Patterns of Antidepressant Medication and Tamoxifen Use Among Patients With Hormone Receptor‐Positive Breast Cancer

**DOI:** 10.1002/cnr2.70608

**Published:** 2026-06-21

**Authors:** Nerea Lopetegui‐Lia, Alexandra Murray, Mariah Ondeck, Bennett Osantowski, Sowmya Takkellapati, Wei Wei, Amanda Maggiotto, Halle C. F. Moore, Erin Roesch

**Affiliations:** ^1^ Division of Medical Oncology, Department of Internal Medicine The Ohio State University Comprehensive Cancer Center Columbus Ohio USA; ^2^ Integrated Surgical Institute Cleveland Clinic Cleveland Ohio USA; ^3^ Department of Internal Medicine Cleveland Clinic Cleveland Ohio USA; ^4^ Department of Hematology/Medical Oncology Taussig Cancer Institute, Cleveland Clinic Cleveland Ohio USA; ^5^ Department of Quantitative Health Sciences Cleveland Clinic Cleveland Ohio USA

**Keywords:** antidepressants, breast cancer, drug–drug interaction, practice patterns, tamoxifen

## Abstract

**Background:**

Some commonly prescribed antidepressants inhibit CYP2D6, an enzyme that metabolizes tamoxifen into its active metabolite, endoxifen. This inhibition could theoretically decrease the efficacy of tamoxifen, which could hypothetically lead to higher breast cancer recurrence rates and mortality. There is inconsistent data reported on the potential drug–drug interaction between concomitant use of tamoxifen and CYP2D6‐inhibiting antidepressants.

**Aim:**

We investigated prescribing patterns of concomitant tamoxifen and antidepressant medications.

**Methods and Results:**

Our study population included adult women ≥ age 18, with stage 0–IV breast cancer who were treated at the Cleveland Clinic, received tamoxifen, and were concurrently prescribed at least 1 antidepressant medication. Patients diagnosed between 2016 and 2021 were identified from a tumor registry database. Among the 405 included patients, 74.8% were taking at least 1 antidepressant before initiating tamoxifen, while 25.2% were started on antidepressant therapy after beginning tamoxifen. Following tamoxifen initiation, 66.3% of patients who had been on an antidepressant continued with the same medication. Among those who switched antidepressants, 16.8% changed to a serotonin‐norepinephrine reuptake inhibitor (SNRI), 12.8% changed to a different selective serotonin reuptake inhibitor (SSRI), and 2.6% changed to another class of antidepressant.

**Conclusions:**

There is awareness of tamoxifen and antidepressant interaction among healthcare providers. However, differences in prescription patterns across providers exist. Individualizing care and, when appropriate, opting for antidepressants with weak or no CYP2D6 inhibition that are equally effective in treating mood disorders or other conditions may be important. While no adherence or outcomes data are presented here, a collaborative, interdisciplinary approach may help support consistent and effective oncologic and behavioral health patient care.

## 1 Introduction

Patients with estrogen receptor‐positive (ER+) breast cancer (BC) treated with tamoxifen for at least 5 years in the adjuvant setting experience significant reductions in risk of BC‐related recurrence and mortality at 15 years, compared to no endocrine treatment (ET) [1]. Furthermore, distant recurrence risk for patients with hormone receptor‐positive BC after 5 years of adjuvant ET persists for another 2 decades, and extended adjuvant ET for up to 10 years is considered for some patients [2]. Tamoxifen is converted into its active metabolite, endoxifen, by the cytochrome P450 2D6 (CYP2D6) enzyme which is inhibited by some antidepressant medications.

Major depressive disorder (MDD) and/or generalized anxiety disorder (GAD) are highly prevalent and disabling conditions among patients with BC [3, 4]. Given this high burden, management of mood symptoms is an important component of cancer care. Antidepressants, particularly selective serotonin reuptake inhibitors (SSRIs) and serotonin‐norepinephrine reuptake inhibitors (SNRIs), are among the most commonly utilized pharmacological treatments for MDD and GAD [3]. In addition to mood disorders, these agents are also frequently prescribed for trauma and stressor‐related disorders and for vasomotor symptoms, often induced by cancer‐directed therapy [5].

Antidepressants within the SSRI class may inhibit CYP2D6 to varying degrees. SSRIs that are strong or moderate inhibitors of CYP2D6 have the potential to interact with tamoxifen through reducing metabolism to endoxifen, which appears to be important for antitumor activity [6]. As a result of either direct or competitive enzyme inhibition [7] reduced CYP2D6 activity can lower endoxifen plasma concentration, which could then theoretically lead to higher risk of cancer recurrence [5].

In response to this interaction, the US Food and Drug Administration (FDA) issued a warning advising against the concomitant use of tamoxifen with strong CYP2D6 inhibitors, including some SSRIs, such as paroxetine and fluoxetine [8], when possible [9]. However, multiple clinical studies investigating the association between concurrent SSRI use and tamoxifen therapy on BC recurrence have yielded heterogeneous and inconclusive results [7]. Some studies reported an increased risk of recurrence, whereas others have found no significant association [5]. Furthermore, the clinical significance of pharmacogenetic factors in tamoxifen effectiveness remains uncertain and incompletely understood [7]. Tamoxifen metabolism may be influenced not only by CYP2D6 activity, but also by other CYP isoforms, CYP2D6 phenotypes, and additional factors such as ABC transporters and broader metabolizer profiles [10, 11].

This controversy leads to differences in prescribing practices across providers and can cause significant distress for patients, particularly those whose mental health concerns have been stable on antidepressant medications. The survival benefit associated with tamoxifen highlights the importance of maintaining patients on ET. It is therefore essential to optimize the management of coexisting mood and vasomotor symptoms to help patients adhere to therapy and achieve the best BC‐related outcomes. To better understand current practice patterns, we evaluated the prescribing of concomitant tamoxifen and antidepressant medications at a large academic institution with affiliated regional practices. Our analysis was specifically focused on tamoxifen and did not include prescribing patterns in patients on aromatase inhibitors (AIs).

## 2 Methods

This is a single‐institution, retrospective study conducted at the Cleveland Clinic Foundation, including the Main Campus in Cleveland, Ohio, as well as affiliated regional sites across Ohio. Our study population included adult women aged 18 to 99 years, with stage 0–IV BC, diagnosed between January 1, 2016, and December 31, 2021, and who received ET with tamoxifen and were concurrently prescribed at least one antidepressant medication during the course of their BC treatment. Patients with stage 0 disease (ductal carcinoma in situ) were also included. Patients with incomplete medical records or missing key treatment data were excluded. Patients were identified from a tumor registry database. Individual patient charts were reviewed by study authors, and relevant clinical, demographic and treatment data were extracted from medical record review and the tumor registry database. Extracted data were securely stored and managed in REDCap.

The treatment regimen included: tamoxifen, which was administered according to standard clinical practice. Antidepressants varied by patient and included SSRIs, SNRIs, and other classes as clinically indicated.

The objectives of this study were to characterize patterns of antidepressant prescribing among patients receiving tamoxifen, and to investigate patient characteristics, medication choices, and prescriber type when these agents are given concurrently. The primary endpoint was the proportion of patients receiving concomitant treatment who were prescribed and maintained on a strong or moderate CYP2D6 inhibitor versus those who were started on or changed to alternative antidepressant medications. Additional endpoints included factors associated with the use of a strong CYP2D6‐inhibiting antidepressants versus other antidepressants, including patient characteristics, prescriber specialty, antidepressant indication, class of antidepressant initiated in patients already receiving tamoxifen, and the frequency of change between antidepressant classes (e.g., discontinuing an SSRI and initiating a SNRI).

We classified antidepressants by degree of CYP2D6 inhibition (Table 1): strong/moderate inhibition (paroxetine, fluoxetine, duloxetine, bupropion, amitriptyline); weak inhibition (citalopram, escitalopram, fluvoxamine, sertraline, nefazodone, buspirone); and those with no identified inhibition (vilazodone, vortioxetine, desvenlafaxine, mirtazapine, trazodone, venlafaxine) [12, 13, 14].

**TABLE 1:** List of medications by level of CYP2D6 inhibition.

Inhibition level	Medications
Strong/moderate	Paroxetine, fluoxetine, duloxetine, bupropion, amitriptyline
Weak	Citalopram, escitalopram, fluvoxamine, sertraline, nefazodone, buspirone
No identified inhibition	Vilazodone, vortioxetine, desvenlafaxine, mirtazapine, trazodone, venlafaxine

*Note:* Buspirone is technically an anxiolytic, not an antidepressant.

The study protocol was approved by the Cleveland Clinic Institutional Review Board (IRB approval #22‐647 DATA1122). As part of this approval, waiver of informed consent was obtained due to the retrospective nature of the study.

### 2.1 Statistical Analysis

Patient characteristics are summarized using median, interquartile range (IQR), and range for continuous variables, and by frequencies and percentages for categorical variables. Antidepressant change status was tabulated against baseline CYP group. Statistical analysis was descriptive and no comparison was made.

## 3 Results

Among patients diagnosed between January 1, 2016, and December 31, 2021, 405 patients met study inclusion criteria and were included in the analysis. Of these, 74.8% (*n* = 303) were taking at least 1 antidepressant medication prior to initiating treatment with tamoxifen, and 25.2% (*n* = 102) were started on an antidepressant after tamoxifen was prescribed.

### 3.1 Patient Sociodemographic Characteristics

Most patients (90.9%, *n* = 368) were White, followed by Black (5.4%, *n* = 22), and < 1% were either Asian, American Indian, or Alaska Native. Most patients identified as non‐Hispanic (97.5%, *n* = 395). Among patients already on an antidepressant at diagnosis, 92.1% (*n* = 279) were White, and 4.3% (*n* = 13) were Black. Within the cohort of patients who were started on antidepressants after tamoxifen initiation, 87.3% (*n* = 89) were White, and 8.8% (*n* = 9) were Black. There were no Asian, American Indian, or Alaska Native patients who were started on antidepressants after tamoxifen initiation in this subset.

Age at BC diagnosis ranged from 23 to 95 years, with a median age of 51 years. Over 80% of patients had an Eastern Cooperative Oncology Group (ECOG) performance status of 0 or 1 at the time of diagnosis. All but 1 patient in the cohort were insured; 60.5% (*n* = 245) had private insurance, 36.5% (*n* = 153) had government insurance, and 6 patients had unknown insurance status. Patients who started an antidepressant prior to versus after tamoxifen initiation did not differ regarding insurance type. Additional details on patient demographics can be found in Table 2A.

**TABLE 2A:** Patient demographics.

	All		Patients taking an antidepressant *after* tamoxifen start (*n* = 102)		Patients taking an antidepressant *prior to* tamoxifen start (*n* = 303)	
	*n*	%	*n*	%	*n*	%
Race						
American Indian or Alaska Native	2	0.5	0	0.0	2	0.6
Asian	3	0.7	0	0.0	3	1
Black or African American	22	5.4	9	8.8	13	4.3
Multiracial	1	0.25	0	0.0	1	0.3
White	368	90.9	89	87.3	279	92.1
Other	8	2	4	4.2	4	1.3
Unknown	1	0.25	0	0.0	1	0.3
Ethnicity						
Non‐Hispanic	395	97.5	98	96.1	297	98.0
Hispanic	6	1.5	2	1.9	4	1.3
Unknown	4	1	2	1.9	2	0.7
Menopausal status at diagnosis						
Unknown	6	1.4	5	4.9	1	0.3
Pre‐Menopausal	208	51.4	46	45.1	162	53.5
Post‐Menopausal	191	47.2	51	50	140	46.2

### 3.2 Breast Cancer Characteristics

Regarding BC histology, 71.8% (*n* = 291) of patients had invasive ductal carcinoma (IDC), 11.1% (*n* = 45) were diagnosed with invasive lobular cancer (ILC), 7.9% (*n* = 32) had mixed histology (ductal and lobular), 1.2% (*n* = 5) had ductal carcinoma in situ (DCIS), and 7.9% (*n* = 32) were identified as “other.” In terms of BC staging, across all patients, 53.3% (*n* = 216) had T1–T2 disease [T1a (*n* = 20), T1b (*n* = 44), T1c (*n* = 70), or T2 (*n* = 82)]. Most patients (84.7%, *n* = 343) had no nodal disease (N0); similarly, in 95% (*n* = 385) of cases, there was no known metastatic disease (M0) at diagnosis. When evaluating the tumor grade, 49.4% (*n* = 200) had grade 2, 24.4% (*n* = 99) had grade 3, and 18.8% (*n* = 76) had grade 1 tumors. Additional details can be found in Table 2B.

**TABLE 2B:** Breast cancer characteristics.

	All		Patients taking an antidepressant *after* tamoxifen start		Patient taking an antidepressant *prior to* tamoxifen start	
	*n*	%	*n*	%	*n*	%
Histology						
Invasive ductal carcinoma	291	71.8	71	69.6	220	72.6
Invasive lobular carcinoma	45	11.1	14	13.7	31	10.2
Mixed invasive ductal and lobular carcinoma	32	7.9	11	10.8	21	6.9
DCIS	5	1.2	2	1.9	3	1
Other	32	7.9	4	4	28	9.2
Clinical T stage						
Unknown	4	1	3	2.9	1	0.33
T0	1	0.25	0	0.0	1	0.33
Tis	144	35.5	42	41.2	102	33.7
T1a	20	4.9	5	4.9	15	4.95
T1b	44	10.9	7	6.9	37	12.2
T1c	70	17.3	21	20.6	49	16.17
T2	82	20.25	13	12.7	69	22.8
T3	14	3.5	6	5.88	8	2.6
T4a	2	0.5	2	1.96	0	0.0
T4b	3	0.7	0	0.0	3	1
T4c	1	0.25	0	0.0	1	0.33
T4d	5	1.2	0	0.0	5	1.65
Other	10	2.45	2	1.96	8	2.6
TX	5	1.2	1	0.98	4	1.3
Clinical N stage						
Unknown	7	1.7	2	1.96	5	1.7
N0	343	84.7	85	83.33	258	85.2
N0 (i+)	27	6.7	8	7.8	19	6.3
N0 (mol+)	7	1.7	1	0.98	6	1.9
N1mi	2	0.5	1	0.98	1	0.33
N1a	2	0.5	1	0.98	1	0.33
N2b	1	0.25	0	0.0	1	0.33
N3b	1	0.25	0	0.0	1	0.33
N3c	1	0.25	0	0.0	1	0.33
Other	9	2.2	1	0.98	8	2.6
NX	5	1.2	3	2.94	2	0.66
Clinical M stage						
Unknown	8	2	3	2.94	5	1.65
M0	385	95.0	98	96.1	287	94.7
M1	4	1.0	0	0.0	4	1.32
Other	8	2	1	0.98	7	2.3
Grade						
Grade 1 (Well differentiated)	76	18.8	21	20.6	55	18.2
Grade 2 (Moderately differentiated)	200	49.4	57	55.9	143	47.2
Grade 3 (Poorly differentiated)	99	24.4	21	20.6	78	25.7
Unknown	30	7.4	3	2.9	27	8.9

### 3.3 Antidepressant Indications

Of the 405 patients, 82.5% (*n* = 334) were prescribed an antidepressant for depressed mood related to a MDD diagnosis; the indication was hot flashes in 9.6% (*n* = 39) of patients. Forty percent (*n* = 162) of patients carried a diagnosis of a psychiatric disorder other than MDD, such as GAD with or without concurrent MDD.

#### 3.3.1 Use of Strong or Moderate CYP2D6 Inhibitors: Paroxetine, Fluoxetine, Duloxetine, Bupropion, Amitriptyline

Of the 90 patients taking strong or moderate inhibitors of CYP2D6 prior to their BC diagnosis, 54.5% (*n* = 49) continued these with tamoxifen treatment (no change) and 43.3% (*n* = 39) of patients changed to a different antidepressant, most commonly an SSRI (24 patients) or SNRI (14 patients); 1 patient changed to an atypical antidepressant. In 2 patients, an SSRI was added to the ongoing regimen for MDD. Among these patients, there were 13 whose treatment plan (prior to BC diagnosis) included a combination of 2 agents (bupropion plus an SSRI or SNRI). An additional 13.7% (*n* = 14) of the 102 patients starting an antidepressant after initiating tamoxifen received a strong or moderate CYP2D6 inhibitor. Overall, 92.9% (*n* = 13) of patients were maintained on an antidepressant that strongly or moderately inhibited CYP2D6 while being treated with tamoxifen, and 1 patient was changed to an atypical antidepressant.

#### 3.3.2 Use of Weak CYP2D6 Inhibitors: Citalopram, Escitalopram, Fluvoxamine, Sertraline, Nefazodone, Buspirone

A total of 135 patients were taking weak CYP2D6 inhibitors prior to their BC diagnosis; 66.7% (*n* = 90) continued these with tamoxifen treatment (no change), and 32.6% (*n* = 44) changed to a different antidepressant (and 1 patient added a second agent to their regimen). Patients most commonly changed to an SNRI (32 patients), followed by an SSRI (8 patients), an atypical antidepressant (3 patients), or a tricyclic antidepressant (TCA) (1 patient). One patient was changed to an atypical antidepressant, and later mirtazapine was added for increased effectiveness.

#### 3.3.3 No Identified CYP2D6 Inhibition: Vortioxetine, Venlafaxine, Desvenlafaxine, Mirtazapine, Vilazodone, Trazodone

There were 78 patients taking an antidepressant with no identified CYP2D6 inhibition: 84.6% (*n* = 66) were taking an SNRI, 8.9% (*n* = 7) an atypical antidepressant, and 6.4% (*n* = 5) serotonin modulators, including 1 patient who was on vortioxetine plus buspirone. About 79.5% (*n* = 62) of the patients who were on these antidepressants prior to tamoxifen start did not change antidepressant class, while 9% (*n* = 7) changed to an SSRI, 7.7% (*n* = 6) to an SNRI, 2.6% (*n* = 2) to a TCA, and 1 to an atypical antidepressant.

Of note, 5 patients stopped their antidepressant medications (mostly SSRIs) prior to starting tamoxifen, and 1 patient who was prescribed multiple psychiatric medications for comorbid psychiatric conditions (including different classes of antidepressants) maintained their existing regimen.

### 3.4 Antidepressant Changes

Overall, about 71.6% (*n* = 290) of patients had no change in class or dose adjustment to their antidepressant. In addition, 3.7% (*n* = 15) discontinued the antidepressant: 6 patients were weaned off prior to starting tamoxifen or within days of initiating ET, 3 discontinued the antidepressant within the first 6 months, 4 stopped due to ineffectiveness, and 2 stopped for unclear reasons. A quarter of the entire cohort (25.7%, *n* = 104) was prescribed an antidepressant that is a strong or moderate CYP2D6 inhibitor, among which 38.5% (*n* = 40) changed to a different class after starting tamoxifen treatment. Interestingly, 29.5% (*n* = 49) of the 166 patients on a weak CYP2D6 inhibitor antidepressant also underwent a change. A total of 135 patients were taking an antidepressant with no identified CYP2D6 inhibition. Of note, there were 17 patients who were on 2 antidepressants and were maintained on these medications.

Most patients who were prescribed an antidepressant prior to or after the initiation of tamoxifen did not change antidepressant class, regardless of the degree of CYP2D6 inhibition (71.6%, *n* = 290). However, for those who did, the distribution was the following: of the patients on a strong/moderate CYP2D6 inhibitor (total of 104 patients), 23.1% (*n* = 24) changed to an SSRI, 13.5% (*n* = 14) changed to an SNRI, and 1.9% (*n* = 2) changed to an atypical antidepressant. Among patients who were taking a weak CYP2D6 inhibitor (total of 166), 21.1% (*n* = 35) changed to an SNRI, 4.8% (*n* = 8) to an SSRI, and 3.6% (*n* = 6) to an atypical antidepressant or TCA. In both cohorts, patients taking agents with strong/moderate or weak CYP2D6 inhibition changed antidepressants due to the thought of having less interaction with tamoxifen or due to lack of efficacy.

Additionally, 28.4% (*n* = 115) changed from tamoxifen to an AI, in some cases due to transitioning from pre‐ to post‐menopausal status, and in other instances due to side effects, including concern for drug–drug interaction.

### 3.5 Patients Who Were Taking an Antidepressant *Prior* to Tamoxifen Start

The main indications for antidepressant use were MDD in 85.8% (*n* = 260) of patients, and hot flashes in 7.6% (*n* = 23). In 69.3% (*n* = 210) of cases, the prescriber of the antidepressant was a primary care physician (PCP, either internist or family physician), followed in frequency of prescribing by a psychiatrist in 10.2% (*n* = 31) of cases. Among this cohort, in 66.3% (*n* = 201) of cases, the antidepressant class was not changed after starting tamoxifen. However, a dose adjustment was made to the antidepressant in 20.8% (*n* = 63) of cases. Among those who underwent a class change (*n* = 99), in 52.5% (*n* = 52) the antidepressant was changed to an SNRI, 39.4% (*n* = 39) to a different SSRI, 5.05% (*n* = 5) to an atypical antidepressant (mostly bupropion), and 3% (*n* = 3) to a TCA. In 31.3% (*n* = 31) of patients, the reason for antidepressant class change was because it was thought to have less interaction with tamoxifen; in 2% (*n* = 2) of cases, it was due to antidepressant side effects. In 40.4% (*n* = 40) of patients, the PCP changed the antidepressant medication, followed in frequency by the medical oncologist in 15.2% (*n* = 15) of cases and a psychiatrist in 11.1% (*n* = 11) patients.

### 3.6 Patients Who Started Taking an Antidepressant *After* Tamoxifen Initiation

Among patients who initiated antidepressants after starting tamoxifen, 64.7% (*n* = 66) were being treated for MDD and 19.7% (*n* = 20) for hot flashes. PCPs prescribed the antidepressant in 55.9% (*n* = 57) of patients, followed by academic and community medical oncologists (9.8%, *n* = 10 and 8.8%, *n* = 9, respectively) and psychiatrists (8.8%, *n* = 9). The antidepressant class change was made by a PCP in 5.9% (*n* = 6) of patients, by a medical oncologist in 5.9% (*n* = 6), and by a psychiatrist in 2.9% (*n* = 3). Additional details on antidepressant and tamoxifen use, as well as practice patterns, can be found in Table 3.

**TABLE 3:** Antidepressant and tamoxifen use practice patterns.

	Patients taking an antidepressant after tamoxifen start (*n* = 102; 25.2%)			Patients taking an antidepressant prior to tamoxifen start (*n* = 303; 74.8%)		
	Strong/Moderate CYP inhibition	Weak CYP inhibition	No identified CYP inhibition	Strong/Moderate CYP inhibition	Weak CYP inhibition	No identified CYP inhibition
Total	14 (13.7%)	31 (30.4%)	57 (55.9%)	90 (29.7%)	135 (44.6%)	78 (25.7%)
No Change	13 (92.9%)	26 (83.9%)	50 (87.7%)	49 (54.5%)	90 (66.7%)	62 (79.5%)
Changed To						
SNRI	0	3 (9.7%)	1 (1.8%)	14 (15.5%)	32 (23.7%)	6 (7.7%)
SSRI	0	0	3 (5.3%)	24 (26.7%)	8 (5.9%)	7 (9%)
Atypical/TCA/other	1 (7.1%)	2 (6.4%)	1 (1.7%)	1 (1.1%)	4 (3%)	3 (3.8%)
Added a second agent	0	0	0	2 (2.2%)	1 (0.7%)	0
Unknown	0	0	2 (3.5%)	0	0	0

### 3.7 Patient Reported Side Effects From Tamoxifen Use

No endometrial cancers or strokes were observed in this cohort of patients, and less than 2% (*n* = 8) developed a deep vein thrombosis. Other documented side effects associated with tamoxifen included hot flashes (40.2%, *n* = 163), night sweats (5.9%, *n* = 24), fatigue (9.4%, *n* = 38), headaches (3.2%, *n* = 13), weight gain (6.7%, *n* = 27), and vaginal dryness and/or discharge (1%–2%).

## 4 Discussion

In patients with ER+ BC, effective compliance with ET is key to achieving optimal disease‐related outcomes. However, full adherence to ET for at least 5 years can be challenging for many patients. Mood concerns are a main factor that could affect maintenance on ET. Depressive symptoms tend to be underreported and undertreated in women with BC.

Our study is descriptive in nature and does not allow for conclusions regarding the safety or efficacy of concomitant use of these agents. These outcomes have been evaluated in prior studies with inconsistent findings. The objective of this study was to assess the level of awareness within our institution, and to better characterize prescribing patterns in the context of this theoretical risk.

Given the importance of both, tamoxifen in BC treatment and antidepressants for managing mental health conditions, several systematic reviews have evaluated whether pharmacokinetic interaction between these agents leads to clinically meaningful effects [15]. Overall, findings have been inconsistent. For example, a systematic review conducted by Bradbury et al. did not show that concurrent use of tamoxifen and antidepressants had a consistent negative effect on clinical outcomes. Most studies included in that review did not demonstrate an increased risk of BC recurrence associated with CYP2D6 inhibitor use [15]. In contrast, Busby et al. reported higher cancer‐related mortality among patients using SSRIs, although the underlying mechanism remains unclear. Preclinical data suggest that SSRIs may increase prolactin levels, a known risk factor for BC progression and a means of facilitating metastasis. Their findings also partially support the hypothesis that CYP2D6 inhibition by SSRIs could contribute to poorer cancer outcomes in patients receiving tamoxifen [4].

Assessing drug–drug interactions for agents like tamoxifen remains challenging, particularly when the potential outcome is suboptimal therapeutic effect that may not become evident for several years. Determining that a drug–drug interaction contributed to therapeutic failure is further complicated by the variability in individual patient response, as some patients may not respond to therapy for reasons unrelated to drug interactions [10]. These factors contribute to ongoing uncertainty in the literature and highlight the difficulty in establishing clear causal relationships.

Overall, it is estimated that about 50% of the 2.4 million BC survivors in the US are prescribed antidepressant medications. Depression in this population, whether preexisting or arising secondary to the distress of cancer diagnosis and treatment, can negatively impact adherence to cancer therapy, quality of life, and treatment outcomes. Notably, the prevalence of depression among patients with BC is 4 times higher than in the general female population [3]; underscoring the importance of appropriately treating both the cancer and mood disorder. Treatment for depressive disorders or other mental health concerns often involves both, pharmacological management with SSRIs or SNRIs, and psychological support provided by integrated oncology care teams [16].

Antidepressant agent selection needs to be carefully considered, as strong or moderate CYP2D6 inhibiting antidepressants could theoretically interfere with the conversion of tamoxifen into its active form, endoxifen, thereby decreasing its efficacy [10]. As noted, although systematic reviews have generally not demonstrated a clear link between CYP2D6‐inhibiting antidepressants and BC recurrence and mortality, it is important to consider that all studies have some methodological limitations, which compromise clinical practice applicability of this finding [17].

As described in our results, prior to the start of tamoxifen, 29.7% (*n* = 90) of patients were on at least 1 strong/moderate CYP2D6 inhibitor, 44.55% (*n* = 135) were on ≥ 1 weak CYP2D6‐inhibiting antidepressant, and 25.7% (*n* = 78) were on ≥ 1 antidepressant without known CYP2D6 inhibition. The most prescribed antidepressants for patients with BC who were on an antidepressant prior to starting tamoxifen were: venlafaxine, escitalopram, sertraline, citalopram, and duloxetine.

Once patients were started on tamoxifen treatment, 66.3% (*n* = 201) who were previously taking an antidepressant remained on the same agent, while 16.8% (*n* = 51) changed to an SNRI, 12.8% (*n* = 39) changed to a different SSRI, and 2.6% (*n* = 8) changed to an atypical/other class of antidepressant. The timing of the change was variable, ranging from 1 month to 2 years, with no clear pattern observed. Although mostly observational given a lack of documentation in the electronic medical record, the timing and underlying reasons for antidepressant change are thought to be diverse. One plausible reason for early antidepressant change was due to concern for drug–drug interaction, whereas a change that occurred after several months could have been due to side effects or a lack of perceived benefit from the antidepressant itself.

Among patients who started an antidepressant after starting tamoxifen, the majority remained on the same agent, with a change seen in 10.8% (*n* = 11) of patients. Interestingly, 11.9% (*n* = 36) of patients who were on an antidepressant prior to initiating treatment with tamoxifen and 4% (*n* = 4) of patients who were started on an antidepressant after starting tamoxifen were changed to a different antidepressant class due to the thought that there could be less drug–drug interaction.

Based on our results, it appears that there is awareness of tamoxifen and antidepressant interaction among healthcare providers (HCPs). However, we did not observe consistent practice patterns across our institution, and the antidepressant changes made due to concern for drug–drug interaction and impact on efficacy suggest variable knowledge and/or acceptance of more recent research. Of note, despite appropriate counseling and being aware of the drug–drug interaction, some patients chose not to change antidepressant agent due to fear of mood destabilization.

Both prior to and after tamoxifen initiation, the main prescriber of antidepressant agents was the patient's PCP, in 79.9% (*n* = 242) and 39.2% (*n* = 40) of cases, which includes both initial prescribing and subsequent changes made to antidepressant agents respectively. We observed that many oncologists, when indicated, encouraged patients to discuss antidepressant changes with their PCP and/or psychiatrist; there was a smaller percentage of patients receiving antidepressant prescriptions from a psychiatrist after starting tamoxifen (5.9%, *n* = 6), whereas 11.9% (*n* = 36) were already following and being prescribed an antidepressant by a psychiatrist prior to tamoxifen initiation. This is an interesting observation, as one would possibly expect more patients to establish care with psychiatry after a BC diagnosis. However, what is not captured is the number of patients who regularly follow with our team of psychologists for psychotherapy and social workers for counseling and psychosocial support. Integration of psychological support in BC survivorship care is an invaluable asset to optimizing patients' psychosocial needs and has been shown to enhance adherence to medications, including ET [15].

### 4.1 Clinical Implications

BC survivors report a higher prevalence of mild to moderate depression, with a lower quality of life. Our findings highlight the importance of a multidisciplinary approach in the care of BC patients and survivors, particularly addressing and treating mental health conditions, which greatly impact overall well‐being.

Differences in practice patterns were identified within our institution and are likely present due to the drug–drug interaction controversy between tamoxifen and some SSRIs and the perception of its clinical impact. A systematic review on concurrent tamoxifen and antidepressant use was unable to demonstrate consistent evidence to support the strongly recommended guideline statements around the avoidance of concurrent therapy [15].

Although clinical evidence regarding the impact of CYP2D6‐inhibiting antidepressants on tamoxifen efficacy is mixed, the potential consequences of underestimating such drug–drug interactions are more serious than those of overestimating them [10, 17]. Available data suggest that CYP2D6 inhibition could reduce tamoxifen metabolism and lower endoxifen concentrations in some patients, indicating the choice of SSRIs should be made carefully, favoring agents with minimal or no CYP2D6 inhibition when possible [10, 17]. Supporting this, data from a multicenter prospective clinical trial found that, citalopram and venlafaxine, considered weak CYP inhibitors, can competitively inhibit 1 or several enzymes in the tamoxifen metabolic pathway, resulting in lower endoxifen concentrations in certain patients [18]. However, the study concluded that coadministration is unlikely to substantially impact clinical outcomes for most patients [18].

We recommend individualizing the care of each patient, and when appropriate, opting for antidepressants with weak or no CYP2D6 inhibition that are equally effective in treating MDD or other conditions. Alternatively, if antidepressants, anxiolytics, or drugs to control vasomotor symptoms cannot be replaced, changing tamoxifen to an AI, if appropriate, could be considered in select cases.

This approach aligns with European Society of Medical Oncology (ESMO) and National Comprehensive Cancer Network (NCCN) guidelines, which advise that patients receiving tamoxifen should avoid strong or moderate CYP2D6 inhibitors [17, 18, 19]. For the management of vasomotor symptoms, non‐SSRI/SNRI options, such as gabapentin, oxybutynin, and fezolinetant, offer comparable efficacy and minimal CYP enzyme inhibition and should be preferentially considered for patients concurrently taking tamoxifen [18].

### 4.2 Study Limitations

This study has some limitations. It utilized a retrospective design, which did not allow for recruitment of a representative sample across factors such as race and ethnicity. Given that our institution is a highly specialized quaternary center, some patients may be referred for a second opinion and/or only receive in‐person care intermittently and continue to follow with their local care team; therefore, their medical records at our institution, including updated medication lists, may be incomplete. Second, given that both agents, tamoxifen and antidepressants, are oral medications, medication compliance is unclear. Moreover, patients may start/stop medications or make dose adjustments without notifying their HCP, which may not be reflected in their medical charts, and conversations between HCPs and patients are not always well captured in the EMR. In addition, while it is plausible that many postmenopausal women were initially treated with an AI and later switched to tamoxifen—potentially due to AI‐related toxicity—and that antidepressant therapy may have been initiated prior to BC diagnosis or while on other endocrine therapies, these details were not captured in our dataset. In our study, we were unable to account for other unmeasured, but potentially relevant, clinical‐psychological variables. Specifically, validated instruments such as the patient health questionnaire (PHQ‐9) and the generalized anxiety disorder assessment (GAD‐7) were not used to assess depressive or anxiety symptoms; instead, assessments were based on documentation recorded by the patient's clinical team. Lastly, our study is limited by the absence of outcome data, such as recurrence‐free survival or overall survival, affecting interpretation of the results.

Nevertheless, we believe that our results describe the practice patterns of tamoxifen and antidepressant use well and accurately, which is the main goal of this study, and highlight an opportunity for education and practice alignment in this space. While the association between prescribing patterns and possible adverse oncologic outcomes was not captured, we believe it is important to consider the theoretical risk and increase awareness.

### 4.3 Conclusions

Concurrent use of tamoxifen and antidepressant medications is common, and the theoretical risk of reduced tamoxifen efficacy with CYP2D6 inhibitors can influence prescribing decisions. Some patients may switch medications, while others remain on their current antidepressant due to concerns about mood destabilization, highlighting the clinical importance of maintaining stability in mental health treatment.

Even without direct outcome data, understanding the current practice patterns is valuable, as it provides insight into how clinicians balance theoretical risk in a real‐world setting and drug–drug interactions with patient‐centered care. Interdisciplinary collaboration, including consultation and working alongside mental health providers, can support patients during antidepressant initiation or adjustments, particularly for those with chronic mood disorders. Our observations reveal variability in practice and an opportunity to enhance guidance, awareness, and standardization in the management of patients receiving both tamoxifen and antidepressants.

## Author Contributions

**Alexandra Murray:** conceptualization, methodology, writing – review and editing. **Mariah Ondeck:** conceptualization, data curation, formal analysis, investigation, methodology, writing – review and editing. **Wei Wei:** conceptualization, data curation, formal analysis, investigation, methodology, writing – review and editing. **Halle C. F. Moore:** conceptualization, methodology, writing – review and editing. **Bennett Osantowski:** conceptualization, data curation, formal analysis, investigation, methodology, writing – review and editing. **Nerea Lopetegui‐Lia:** conceptualization, data curation, formal analysis, investigation, methodology, writing – original draft, writing – review and editing. **Sowmya Takkellapati:** conceptualization, methodology, writing – review and editing. **Erin Roesch:** conceptualization, methodology, writing – review and editing. **Amanda Maggiotto:** conceptualization, data curation, formal analysis, investigation, methodology, writing – review and editing.

## Funding

The authors have nothing to report.

## Ethics Statement

The study was approved by the Cleveland Clinic Institutional Review Board (IRB approval #22‐647 DATA1122).

## Consent

As part of this approval, waiver of patient consent was obtained due to the retrospective nature of the study.

## Conflicts of Interest

Nerea Lopetegui‐Lia served on advisory board for Pfizer (2026), and received honoraria from Stemline Therapeutics, Curio Science, MJH Life Sciences, and OncLive. This funding was not related to this study. Sowmya Takkellapati served on advisory boards for Novartis (2023) and Gilead (2024), unrelated to this study. Halle CF Moore has research funding paid to the Cleveland Clinic Foundation from AstraZeneca, Daiichi‐Sankyo, Sermonix Pharmaceuticals, Seattle Genetics, and Pfizer, which was not related to this study. Erin Roesch has served on advisory boards for Biotheranostics Inc., Neogenomics, and Onviv Inc., received speaker fees from MJH Life Sciences, OncLive, and Decera Clinical Insights, and received consulting fees from AstraZeneca and Gilead. This funding was not related to this study. The other authors declare no conflicts of interest.

## Data Availability

The data that support the findings of this study are available from the corresponding author upon reasonable request.
